# Overview of the different personalized total knee arthroplasty with robotic assistance, how choosing?

**DOI:** 10.3389/fsurg.2023.1120908

**Published:** 2023-03-03

**Authors:** Mina Wahba Morcos, David Uhuebor, Pascal-André Vendittoli

**Affiliations:** ^1^ Department of Surgery, Hôpital Maisonneuve-Rosemont, Montreal University, Montreal, QC, Canada; ^2^ Clinique Orthopédique Duval, Laval, QC, Canada; ^3^ Personalized Arthroplasty Society, Atlanta, GA, United States

**Keywords:** knee - surgery, arthroplasty, kinematic, alignment, robot, personalized, function

## Abstract

Current limitations in total knee arthroplasty (TKA) function and patient satisfaction stimulated us to question our practice. Our understanding of knee anatomy and biomechanics has evolved over recent years as we now consider that a more personalized joint reconstruction may be a better-targeted goal for TKA. Implant design and surgical techniques must be advanced to better reproduce the anatomy and kinematics of native knees and ultimately provide a forgotten joint. The availability of precision tools as robotic assistance surgery can help us recreate patient anatomy and ensure components are not implanted in a position that may compromise long-term outcomes. Robotic-assisted surgery is gaining in popularity and may be the future of orthopedic surgery. However, moving away from the concept of neutrally aligning every TKA dogma opens the door to new techniques emergence based on opinion and experience and leads to a certain amount of uncertainty among knee surgeons. Hence, it is important to clearly describe each technique and analyze their potential impacts and benefits. Personalized TKA techniques may be classified into 2 main families: unrestricted or restricted component orientation. In the restricted group, some will aim to reproduce native ligament laxity versus aiming for ligament isometry. When outside of their boundaries, all restricted techniques will induce anatomical changes. Similarly, most native knee having asymmetric ligaments laxity between compartments and within the same compartment during the arc of flexion; aiming for ligament isometry induces bony anatomy changes. In the current paper, we will summarize and discuss the impacts of the different robotic personalized alignment techniques, including kinematic alignment (KA), restricted kinematic alignment (rKA), inverse kinematic alignment (iKA), and functional alignment (FA). With every surgical technique, there are limitations and shortcomings. As our implants are still far from the native knee, it is primordial to understand the impacts and benefits of each technique. Mid to long data will help us in defining the new standards.

## Personalized TKA, a new ERA

The complexity of the knee joint was made more evident by recognizing the patients’ symptoms, kinematic changes, and dysfunctions related to the anatomical modifications produced by the traditional systematic total knee arthroplasty (TKA) surgical techniques. Nowadays, such understanding leads to redefining surgical goals towards a personalized surgical approach. With robotic assistance availability, several new techniques are proposed as alternatives to the one size fits all concept. However, as none of these techniques bear reliable scientific data to be considered the new gold standard, it is essential for the new users to understand their differences and for the scientists to undertake high-quality comparative studies. The current paper will summarize and discuss the impacts of the different robotic personalized alignment techniques.

The normal anatomy varies widely, and this variability is further increased in the presence of pathological processes (1). The TKA mechanical alignment (MA) technique is widely employed and has a systematic implant placement without considering the native knee anatomy. This technique was considered the “gold standard” as it was a more straightforward and reproducible method in an era where precision tools were unavailable. On the other hand, it introduces significant anatomic modifications, ligament imbalances, and suboptimal kinematics, leaving the knee feeling less natural (2, 3) and a high dissatisfaction rate (4).

Therefore, the understanding of native knee anatomy and biomechanics is cardinal, and its application has improved over recent years. Lower limb native coronal alignment demonstrates considerable individual variability, making one-size-fits-all comers notably less suitable. This is pointedly evident in a study evaluating 4,884 pre-operative lower limb computed tomography (CT) scans of patients undergoing TKA showed only 5% of the femur and 4% of the tibia had neutral joint line orientation with only 0.1% of the knees had both tibia and femoral joint lines in neutral orientation (5, 6). The goal of the MA technique is a neutral orientation, and this functional knee phenotype target was found in only 3.6% of females and 5% of males (7). Such variable individual joint surfaces’ orientations lead to specific knee kinematics. Eckhoff et al., in their work, defined the three kinematic axes which dictate the motion of the tibia and patella around the femur. The condylar or cylindrical axis between 10° and 120° of knee flexion is the locus about which the tibia extends as well as flexes, and not the trans-epicondylar axis as previously thought (8, 9).

**Figure 1:**
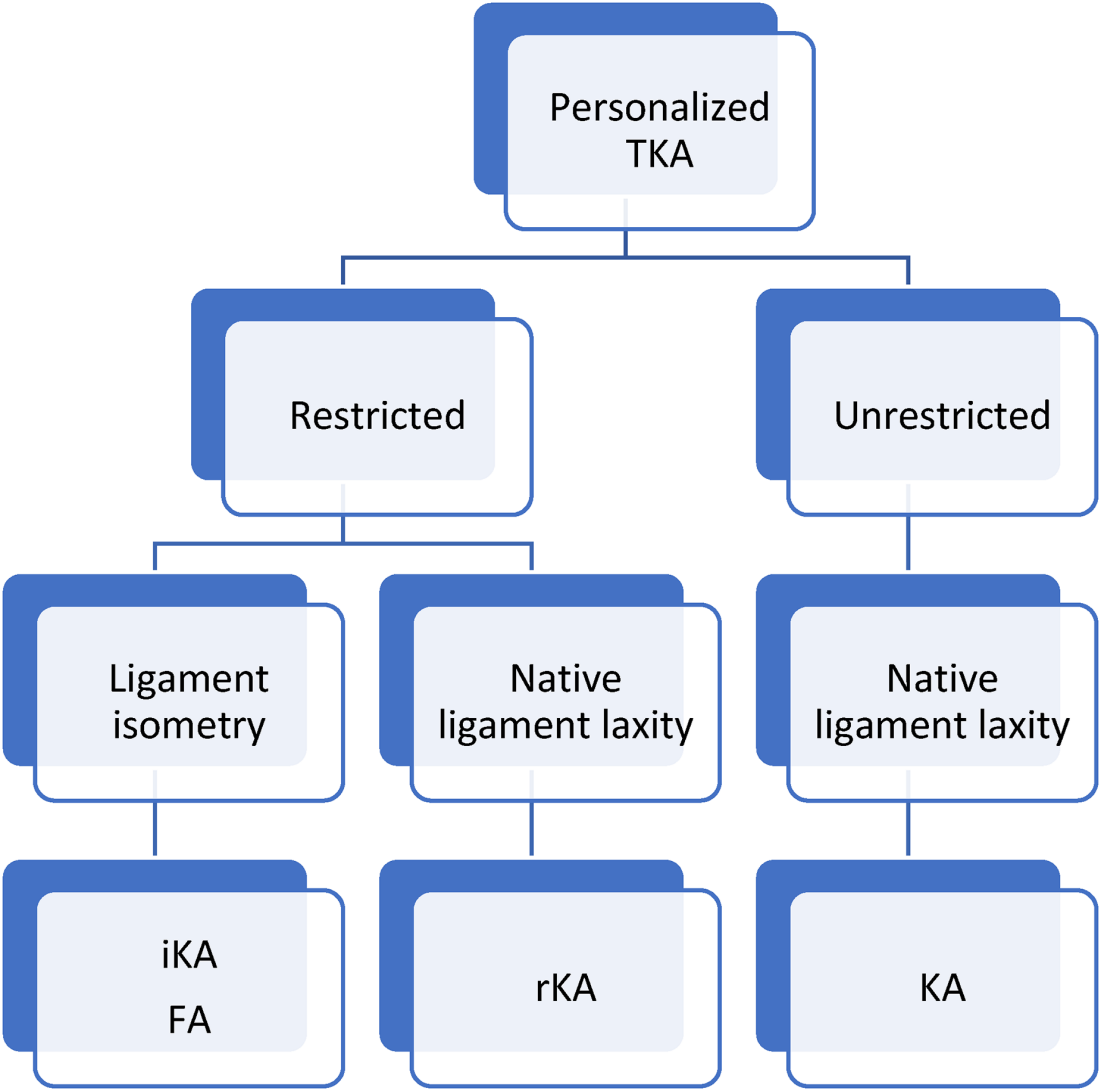
Diagram of the most common personalized total knee replacement surgical techniques. KA, kinematic alignment; iKA, inverse kinematic alignment; FA, fonctional alignment; rKA, restricted kinematic alignment.

In the last decade, Stephan Howell proposed a novel approach to TKA called kinematic alignment (KA) (10). This technique is a true resurfacing of the knee joint, aiming at reproducing the pre-arthritic knee's kinematic axes. Moving away from the systematic MA era is the birth of Personalized TKA (11). Personalized TKA considers the individual's native knee anatomy and physiological soft tissue laxity whilst aiming to produce more natural knee joints and attain patient satisfaction and a forgotten joint (1). On the other hand, outlier anatomies are suspected to be inherently biomechanically inferior and potentially incompatible with current implant material and fixation methods (12). Keeping in mind the historical impact of outlier alignments on TKA survivorship, Vendittoli proposed the application of some alignment boundaries to KA, named restricted KA (rKA) (13). Therefore, Personalized TKA can be categorized into 2 categories: unrestricted and restricted component orientation (Figure 1). Unrestricted includes KA, which aims to reproduce the native ligament laxity. While restricted alignment includes rKA, inverse kinematic alignment (iKA) and functional alignment (FA). Some restricted techniques aim to obtain native ligament laxity, while others aim for ligament isometry.

The choice of implant design in personalized alignment TKA still controversial. Howell et al., showed in his 10-year retrospective study that implant survival rates between KA and MA TKA were comparable using cruciate retaining implants (14). On the other hand, Sappey-Marinier et al. showed an increased risk of aseptic loosening in a case-control comparing rKA to MA using posterior-stabilizing (PS) implants (15). Scott and Grey as well in a randomized control trial using KA technique found less favourable outcomes with PS implants which was attributable to mid-flexion instability (16). It appeals to the mind to think that an implant that better replicates the kinematics of the healthy knee would be more favourable to personalized alignment techniques. In normal knees, it has been shown that as the normal knee goes into flexion, the medial femoral condyle pivots as the lateral slides posteriorly on the tibial plateau resulting in femoral “roll back” motion (17). Medially stabilised implant attempts to replicate this by having a more congruent medial compartment reproducing the physiologic “medial pivot” and a less conforming lateral compartment that permits more motion. As such this physiological movement is reproduced (18). The clinical benefit of this design was shown by Risitano et al. and French et al., comparing the outcomes of a CR and medial stabilized TKA, with both utilizing KA method had better functional outcomes scores as well as forgotten joint scores with the use of medially stabilised TKA (19, 20).

## Robotics assistance

In order to achieve any of these personalized techniques, accuracy, precision and consistent needs and nowadays, robotic assistance availability makes these techniques accessible (21). This allows accurate lower limb alignment and bespoke soft tissue balancing information, thus facilitating precise component sizing, placement and prevents implantation in positions that may compromise long-term outcomes (22). This is clearly at variance with conventional cutting guides, which have been shown to be associated with an increased risk of deviation from planned resection. As much as 30% of knees using conventional guides will have errors >3° off target (23).

Robotic systems can be image-guided, requiring preoperative CT-Scan or MRI to build a 3D plan to template component size and positioning. This 3D model will then be linked to intraoperative anatomical landmarks. Imageless platforms are also available, creating the 3-D model from an intraoperative bone morphology mapping. The image-guide or imageless systems allow virtual and dynamic gap measurement over the knee arc of motion. Such tools will help:

- Precise bone ressection, restoring native soft tissue laxities for pure KA.
- Adjusting bone ressections when rKA boundaries require anatomical odifications and evaluating the resulting gap modifications
- Modifying the femoral and/or tibial bone cuts to achieve ligament isometry with iKA and FA, obviating the need for soft tissue releases.

Robotic systems for knee arthroplasties are further categorized into passive, semiautonomous and autonomous systems based on surgeon control over them. Passive systems provide a 3D virtual model that the surgeon performs guide tool positioning and bone removal. The robot in the autonomous system holds the cutting tool and makes the femoral and tibia resections. The semiautonomous system can be regarded as a combination of both principles; here the surgeon holds control over the bone resections whilst the robot provides real time intraoperative feedback limiting surgeon's action within a safe zone (24, 25).

A meta-analysis of level 1 evidence comparing the four techniques showed that robot and navigation were notably better than PSI and conventional in control of lower limb alignment and component position, with the robot having the lowest probability of outlier (26). Although Kayani et al. (27) reported increased operative times with the implementation of robotic-arm-assisted TKA for the initial seven cases, there was no learning curve for achieving the planned implant positioning and alignment. Notably, using computer navigation to improve TKA alignment precision did not result in clinical improvements with a MA (28). When assessing the performance of precision tools such as computer navigation or robotic assistance, it is crucial to differentiate the terms “precision” and “accuracy” (21). Improved precision provided by these tools is valueless when aiming at the wrong target. Undoubtedly, greater accuracy in TKA surgery is warranted through a more individualized approach. In the following sections, we will describe the most common personalized TKA techniques performed with robotic assistance (KA, rKA, IKA and FA), focusing on their main differences.

## Kinematic alignment (KA)

KA is an unrestricted patient specific TKR technique that more closely replicates the native knee anatomy and soft tissue laxities (6). The knee is resurfaced with the restoration of the pre-arthritic anatomy and maintenance of soft tissue envelope and ligamentous tension KA does not restrict the patient's anatomy or final correction compared to the other alignments. The amount of bone and cartilage removed mirrors the implant thickness restoring the pre-disease knee joint orientation (9). Although Howell showed that the caliper technique learning curve is short and reproducible, robotic assistance remains a powerful tool allowing the surgeon to perform KA with excellent control (29, 30).

Robotic-assisted KA follows the same sequence as calipered KA; firstly, a distal femur cut is made parallel to the joint line after correcting for estimated bone loss. This is followed by a posterior femoral cut performed parallel to the posterior condylar plane. Next, the tibia cut is also made parallel to the joint line, having also corrected for wear. Taking in account the cartilage and bone loss, all resected surfaces must correlate in thickness to the TKA component. Even if robotic assistance is used, determining the amount of bone resected with a caliper can be performed as a safety check (9).

In most knees, the space is nearly rectangular in full extension. Robotic gap measurement tool facilitates such evaluation. In the presence of femorotibial soft tissue imbalances (tightness or excessive laxity) in full extension, it is addressed by tibia bone cut adjustment; no soft tissue release is done (10). Such tibial cut adjustment is easily performed with robotic assistance. With the gap measurement tool, one should ensure that both compartments become looser in flexion, with the lateral compartment looser than the medial (as it is for most native knees).

## Restricted kinematic alignment (rKA)

The rKA fundamentals include five principles (i) Combined lower limb coronal orientation of ±3° of neutral; (ii) Joint line orientation coronal alignment should be within 5° of neutral; (iii) Natural knee's soft tissue tension/laxities preservation/restoration; (iv) Femoral anatomy preservation over tibia; (v) The most intact knee compartment should be resurfaced with a thickness that is equal to the width of the implant and used as pivot point when anatomical adjustment is required (13). rKA requires a precision tool such as a robotic system that provides surgeon patient's anatomy values and allows adjustments when outside the safe boundaries (13).

**Figure 2:**
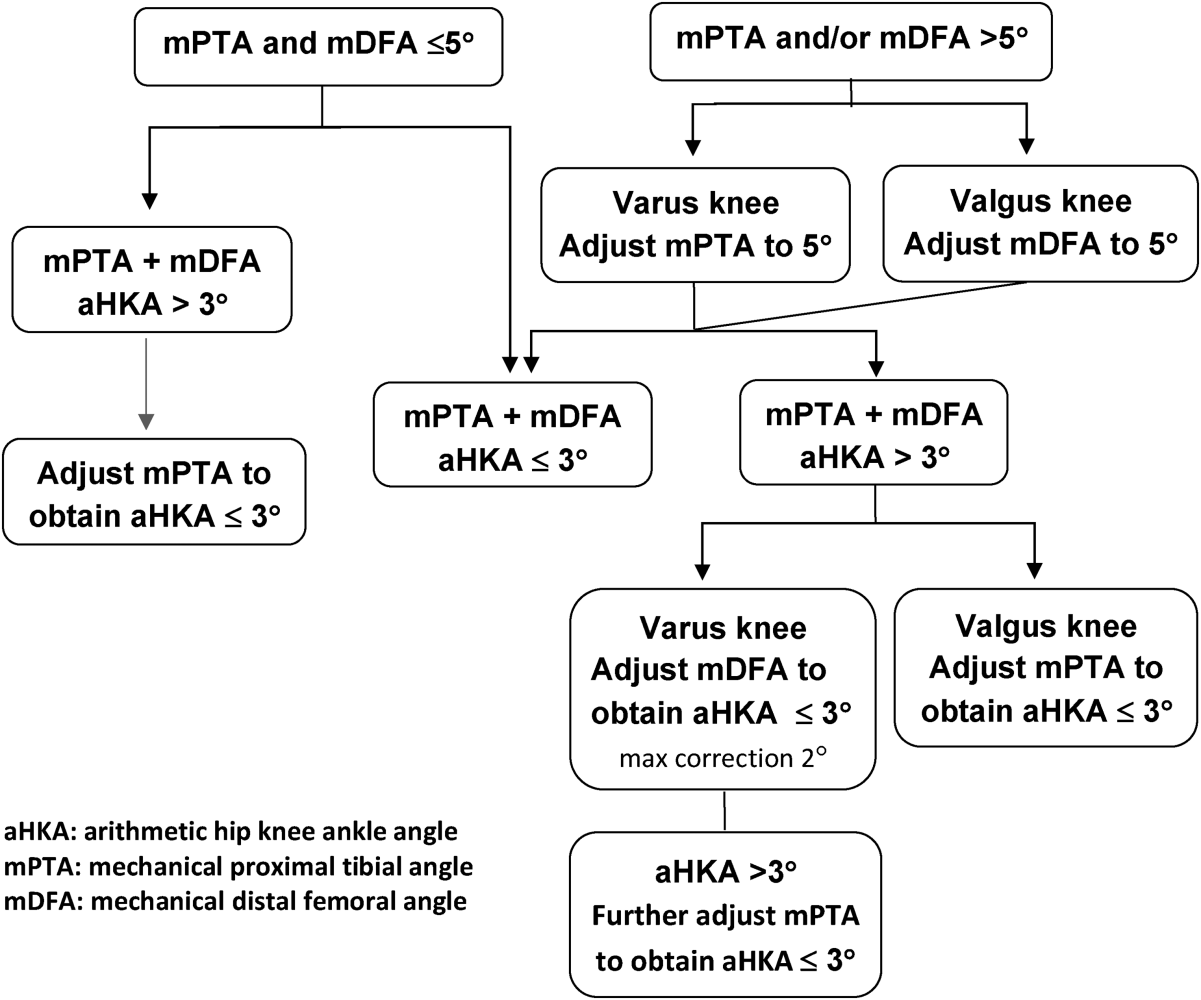
rKA algorithm as proposed by Vendittoli (6, 13).

The knee joint is exposed using a standard approach but preserving the deep medial collateral ligament (MCL) attachment. With the aid of a robotic system, cartilage and bone loss thickness are estimated by comparing it to the intact areas- intact cartilage = 0 mm, partial cartilage thickness wear = 1 mm, and exposed subchondral bone = 2 mm. Next, distal femoral and proximal tibial resections are set at each implant's thickness. On the planning screen, the surgeon should follow the rKA algorithm (Figure 2). If the femoral and/or tibial joint surface orientation fall outside the 5 degrees limit, keeping the resection thickness equal to the implant thickness on the unworn side (example: lateral for varus knee), the cut angle is adjusted to reach the desired angle, reducing the resection thickness on the worn side. In 50% of the cases, patients’ anatomy will fall within the rKA boundaries, and true KA will be performed without adjustment. In 30% of the cases, minor adjustments are needed (<1 degree); in the last 20%, more important modifications will be required.

As for KA, the goal is to restore the native ligament laxities. In most knees, the space is near rectangular in full extension, and both compartments become looser in flexion, with the lateral compartment looser than the medial. When a patient's anatomy adjustment of >2 degrees/2 mm is performed, the created gap modification may require limited soft tissue release. For example, a simple deep MCL release should balance the gaps in most varus knees outside the rKA boundaries. The posterior condyles are resurfaced with the posterior reference guide set to neutral rotation. Keeping the knee in 10° of flexion the tibial component rotation is set by its alignment with the femoral trial component (13). A detailed description of the rKA robotic assisted technique was published by Massé et al. (31).

## Inverse kinematic alignment (iKA)

As a more recent rKA technique, iKA main difference with rKA lies in its ligament isometry goal. A robotic system with a gap-balancing tool is necessary to achieve precise gap adjustment (9). iKA applies restriction in the HKA in a safe zone between 6° varus and 3° valgus, and in order to recreate the native medial proximal tibial angle, a boundary of 6° varus to 2° valgus is applied (9). iKA is a tibia resection first resurfacing technique that restores the pre-articular joint line obliquity after correcting for wear. After the tibial resection, its new surface will be used as the reference for medial and lateral gap balancing in flexion and extension. Gap equalization is achieved by adjusting the distal femoral and posterior resection angulation and thicknesses. As rectangular spaces are obtained, no soft tissue releases are performed, except outside the maintained iKA boundaries: HKA >6° varus (medial release) and >3° valgus (lateral release). In contrast to KA and rKA, iKA uses an external rotation of the femur to obtain a rectangular flexion space (32, 33).

## Functional alignment (FA)

Various authors recently proposed FA as a technique aiming to reconstruct the 3-dimensional (3D) constitutional alignment of the knee, whilst maintaining the adapted soft tissue envelope with the aid of a robotic system (34, 35). This ensures reproducible goals and reduces the risk of missed targets and catastrophic outliers (9). The robotic platform should allow for real-time 3D feedback on flexion and extension gaps and implant positioning and alignment suited to the patient's individual native ligament balance and bony anatomy as well as joint line restoration (2, 36). FA differs from other personalized TKA techniques in that it has defined targets for joint height, obliquity and balanced gaps throughout the range of motion with objective soft tissue laxity endpoints (35).

Functional alignment begins with 3D imaging from pre-operative CT-scan or MRI to aid planning in all planes to determine and improve implant sizing and positioning. Granted, a mechanical or kinematic alignment start point can be chosen, FA with KA starting plan is more likely to achieve all stated goals of FA, especially with regards to restoring joint line plane and obliquity (37). FA philosophies have safe boundaries within which modifications are attempted to achieve a balanced knee. The described component alignment boundaries are: HKA of 6° varus to 3° valgus; femoral component at 6° valgus to 3° varus; tibial component at 6° varus to 3° valgus; combined component flexion of 10° and femoral rotation of 6° valgus to 6° varus from the surgical epicondylar axis (35). With the aid of a robotic system, distal femur resection is made to match the mechanical lateral distal femur angle, which preserves the joint line obliquity. The proximal tibia cut is executed in the coronal plane to align with the mechanical proximal tibial angle, and the posterior tibial slope is in line with the medial plateau to match the patient's native posterior slope in the sagittal plane. On the axial plane, the tibia component is implanted using the line of Akagi. The distal femur and proximal tibial resection depth are performed to match the implant thicknesses with a combined gap difference of no more than 2 mm (9, 35). Pre-emptive soft tissue balancing of flexion and extension gaps to achieve equal mediolateral soft tissue tension is planned prior to bone cuts using the robotic platform. The implants’ position and orientation should mirror the patient's knee through a full range of motion (35, 38).

## Discussion: comparing the different techniques

The value of robotic-assisted surgery may be unlocked by personalized alignment goals as this is geared at the individual patient and restoration of their native anatomy. Although the different personalized alignment techniques: KA, rKA, iKA, and FA aim to respect the patient's phenotypes, significant differences exist between them (Table 1).

**Table 1:** Summary table of personalized TKA techniques.

	KA	rKA	iKA	FA
**Femoral component flexion**	Target: 2° ± 3° of flexion	Target: 2° ± 3° of flexion	Target: 2° ± 3° of flexion	Target: 0°–5° of flexion
**Femoral distal cut**	Parallel to distal femoral joint line (considering wear)	Parallel to distal femoral joint line (considering wear) If > 5°, correct to 5°	Orientation determined by the tibial cut. Goal is to obtain a rectangular extension space	Parallel to distal femoral joint line (considering wear) Target: 0°–5°
**Femoral condyle posterior cut**	Parallel to the posterior condylar line	Parallel to the posterior condylar line	Orientation determined by the tibial cut. Goal is to obtain a rectangular flexion	Surgical transepicondylar axis; ± 3°
**Tibial component coronal cut**	Parallel to proximal tibial joint line (considering wear)	Correct to < 5°, then parallel to proximal tibial joint line (considering wear)	Parallel to proximal tibial joint line (considering wear) within safe zone of 6° varus to 2° valgus	Perpendicular to the tibial mechanical axis
**Tibial slope**	Parallel to the medial plateau slope Target: −6° to 9°	Parallel to the lateral plateau slope Target: < 6°	Parallel to the medial plateau slope Target: −6° to 2°	Parallel to the medial plateau slope Target: 0°–3°
**Tibial rotation**	Parallel to lateral plateau long axis	Aligned with the femur in extension	Parallel to lateral plateau long axis	0°–5° of external rotation to Akagi's line
**Knee balancing goal**	Native ligaments laxities Tibial cut if needed	Native ligaments laxities. Soft tissue releases if needed.	Ligament isometry Femoral cut adjustments (distal and posterior)	Ligaments isometry Femoral and tibial positioning + soft tissues
**Soft tissue release**	None	rarely	sometimes	sometimes
**Technologies**	Robotic-assisted, navigation, PSI, caliper	Robotic-assisted, navigation, PSI	Robotic-assisted	Robotic-assisted

KA favours bony anatomy preservation and native soft tissue laxity restoration. Although Howell et al., at 10 years, obtain a low revision rate with KA TKA (14), concerns remain in the absence of independent corroboration and long-term data. There is a reluctance to reproduce extreme knee anatomies, which may be native or the results of knee trauma, childhood deformity, previous surgery or tumours on account of their inherent biomechanical weakness. As these outliers anatomies may be unsuitable with current prostheses fixation methods and materials, adverse consequence on their long-term survival (6, 13).

For these reasons, rKA was proposed by Vendittoli et al. Restricted KA uses the same KA's technique when there is no significant pathoanatomy while slightly adjusting in more severe cases by aiming for a “safe zone”. rKA is similar to KA, but when outside a safe zone will require bone cut adjustment and soft tissue release. With robotic planification screen, the surgeon can apply the rKA algorithm (Figure 2) to estimate the gap modification imposed by the rKA boundaries, and anticipate the soft tissue release to be performed. A TKA surgical simulation of 1,000 lower limb CT-Scans showed that significantly fewer imbalances are created using rKA versus MA (39). These results were corroborated clinically in a RCT by MacDessi et al., where they found less anatomic modification and need for soft tissue releases in rKA vs. MA (40). Although the rKA technique makes sense for the more conservative surgeons not ready to adopt unrestricted KA, the need for further study to determine the real limits of safe boundaries are still pending, and the current safe zone may certainly change over time.

It is safe to say the ultimate goal of TKA is a natural feeling or forgotten joint. The most frequent reasons patients report for joint awareness or unnatural joint perception were pain followed by reduced knee flexion (41). To date, as well as in a recent meta-analysis that included 1,112 cases, comparing clinical outcomes of KA- 559 cases and MA- 553 cases in TKA. KA TKA showed better knee society scores (KSS) in the KA than the MA group. The Western Ontario and McMaster Universities Osteoarthritis index (WOMAC), higher range of motion of flexion was better in the KA group although Oxford Knee Score (OKS) was similar as well as the complications in both groups. In essence, KA TKA over MA TKA group has better functional outcomes including a greater flexion range of motion as well as better patient satisfaction (42).

In counterparts to KA and rKA, iKA and FA aim for isometric gaps in extension and flexion. As native knee ligament laxities are known to be asymmetric, iKA and FA will modify femoral bony anatomy and flexion axes in most cases. This was clearly shown by Winnock de Grave et al. (32) in a publication reporting the femoral resections thicknesses of varus knees. To compensate for the lateral gap increased laxity versus the medial side, they distalized the lateral condyle by a mean of 1.4 mm and externally rotated the femoral condyle by a mean of 1.7 mm. Such lateral condyle overstuffing was recognized as detrimental for the patella-femoral joint with MA TKA (43). Another key issue in soft tissue balance is mediolateral ligament asymmetry and its influence on total knee arthroplasty outcomes. Although with traditional MA it was prescribed that medial and lateral soft tissue tension be equal in extension and flexion after bone cuts. This is at variance with the behaviour of the native knee in which lateral laxity is known to be greater than medial laxity, particularly in flexion, whereby lateral laxity is cardinal for the medial pivot movement of the normal knee (44). A study on the clinical significance utilized a computer-assisted navigation system to evaluate gap laxities and differentials. It showed that lateral flexion gap laxity is consistently associated with better patient-reported outcome measures (45).

## Conclusion

Keeping in mind that the current implant designs are far from a native knee (morphology, tissue qualities, cruciate ligaments deficient, etc.) it is still unclear if we should restore all anatomies and native ligament laxities. We are now in a transition phase. Whether one personalized robotic assisted TKA technique is superior to the other or provides better patient outcomes is yet unclear, and further studies are needed. In addition, having such a powerful and precise tool as robotic assistance requires deeper comprehension to master the benefits and downsides of these newer approaches (21, 46).
